# Synthesis of Uniform Polyaniline Nanofibers through Interfacial Polymerization

**DOI:** 10.3390/ma5081487

**Published:** 2012-08-22

**Authors:** Ahmad Abdolahi, Esah Hamzah, Zaharah Ibrahim, Shahrir Hashim

**Affiliations:** 1 Faculty of Mechanical Engineering, Universiti Teknologi Malaysia, Johor 81310, Malaysia; E-Mail: Ahmadabdolahi25@yahoo.com; 2 Faculty of Bioscience and Bioengineering, Universiti Teknologi Malaysia, Johor 81310, Malaysia; E-Mail: zaharah@fbb.utm.my; 3 Faculty of chemical engineering, Universiti Teknologi Malaysia, Johor 81310, Malaysia; E-Mail: shahrir@cheme.utm.my

**Keywords:** polyaniline, nanofiber, interfacial polymerization, FESEM, XRD, FTIR

## Abstract

The present paper aims to study the preparation of polyaniline nanofibers through simple interfacial polymerization. Ammonium persulfate, hydrochloric acid and chloroform were used as oxidant, dopant and organic solvent respectively. Field Emission Scanning Electron Microscopy (FESEM), X-ray diffraction and Fourier Transform Infrared Spectroscopy (FTIR) techniques were used to analyze the product. FESEM results show that polyaniline has nano-fiber morphology. XRD results show the crystalline properties of polyaniline nanofiber, and FTIR results confirmed the formation of polyaniline in different monomer/oxidant molar ratios. This study provides a better understanding on the synthesis of uniform polyaniline nanofibers through interfacial polymerization.

## 1. Introduction

Conductive polymers belong to an interesting group of polymers, which have unique electrical, electrochemical and optical properties [1,2]. They could be used in different applications such as hydrogen storages [3,4] sensors [5], solar cells [6], antistatic coatings [7], diodes [8] and anticorrosive coatings [9]. Among conductive polymers, polyaniline has gained more attention due to its economical price, good redox properties and high environmental stability [10]. Polyaniline and its derivatives are synthesized through two general routes: (a) electrochemical; and (b) chemical methods [11]. The common method for fabrication of polyaniline in large quantities is based on chemical methods. In conventional chemical methods, the aniline is polymerized in water based solution in the presence of oxidant and dopant. The synthesized polyaniline appears in the form of irregular granular particles. Several studies have shown that granular polyaniline particles have poor solubility and processability in common solvents such as water [12]. More attempts were made to improve their processability and solubility. It has been found that nanomaterials owing to their high surface area have better solubility in common solvents [13]. 

Different methods used to fabricate polyaniline nanofibers include electrospinning [14], ultrasonic irradiation [15], hard templates [16], soft template [17], interfacial polymerization [18] and seeding polymerization [19]. Among these methods, interfacial polymerization has achieved more attention due to its easiness, large-scale production-ability and environmentally benign nature. Furthermore, it has a capability to produce high-quality polyaniline nanofibers with control of their morphology, size and diameter [18]. Generally, different parameters could influence on the morphology of polyaniline nanofiber. For example, strong acids result in production of high-quality nanofibers with a larger diameter. [20,21]. Another important parameter which could affect the morphology of polyaniline nanofibers is the aniline/oxidant ratio. This paper aims to study the production of polyaniline nanofibers at different aniline/oxidant ratios through simple interfacial polymerization. FESEM, XRD and FTIR techniques were used to analyze the polyaniline nanofibers.

## 2. Experimental

### 2.1. Materials

Aniline monomer, ammonium persulfate, chloroform and hydrochloric acid were purchased from a chemical company and they were used as received without further purification process. 

### 2.2. Synthesis

In conventional synthesis method, 4mmol aniline was dissolved in 20 mL hydrochloric acid (1 M) and stirred with magnetic stirrer for one hour. 4mmol ammonium persulfate was also dissolved in 20 mL hydrochloric acid (1 M) and magnetically stirred for one hour. Then the ammonium persulfate solution was added into the aniline solution and was left at room temperature for 6 hours. Finally, the black green precipitate was collected and washed several times by acetone and distilled water. The pure polyaniline was then dried at ambient temperature for 24 hours. 

In the interfacial polymerization, two types of solutions were prepared: (a) 20 mL hydrochloric acid (1 M) which contains 4 mmole ammonium persulfate; and (b) 20 mL chloroform which contains 4 mmole aniline. Each solution was stirred with magnetic stirrer for one hour. Then the oxidant based solution was carefully transferred to aniline based solution. After a short induction time, the reaction took place and the polyaniline was initially formed at the interface of the immiscible solutions. The green polyaniline nanofibers increased in amount over time and filled the water based solution. After 24 hours, the black green polyaniline nanofibers were filtered and dried at room temperature for 24 hours. 

### 2.3. Characterization

The morphology of polyaniline nanofibers was characterized using the field emission scanning electron microscopy (FESEM, Zeiss-LEO Model 1530). The structures of polyaniline (PANI) were analyzed using Fourier Transform Infrared Spectroscopy (VERTEX70 infrared spectrometer (using pellets with KBr)). The crystalline properties of nanofibers were measured by using X-ray Diffraction (Ultima III X-ray diffractometer with Cu Kα radiation).

## 3. Results and Discussion

### 3.1. Reaction and Mechanism

**Figure 1:**
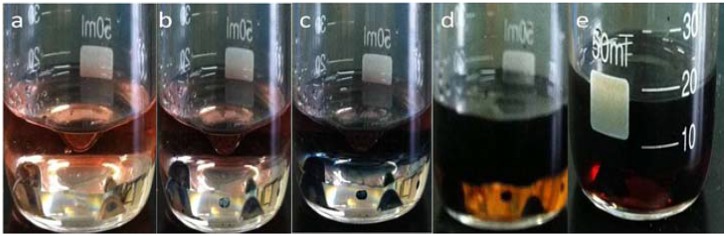
Interfacial polymerization of polyaniline (PANI) nanofiber at (**a**) 5s; (**b**) 20s; (**c**) 1min; (**d**) 5min; (**e**) 1h.

As shown in Figure 1, in interfacial polymerization, the polymerization was performed in an immiscible organic/aqueous system, to separate the byproducts (inorganic salts, oligomers, *etc*.) according to their solubility in the organic and aqueous phases. After a short induction period green PANI appears at the interface, moving into the water phase and finally filling the whole water layer (Figure 1e). As the reaction continues, the color of the organic phase becomes darker and finally stops changing, indicating reaction completion.

**Figure 2:**
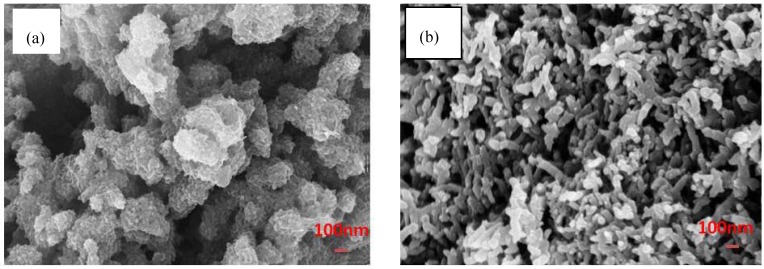
Field Emission Scanning Electron Microscopy (FESEM) image of (**a**) granular PANI synthesized by conventional method; (**b**) PANI nanofiber synthesized by interfacial polymerization.

Figure 2a shows the granular polyaniline which was synthesized through conventional method. In conventional method the oxidant based and aniline based solution was mixed and the new solution was magnetically stirred for 24 hours. The resultant product is irregular polyaniline particles with high agglomeration. 

**Figure 3:**
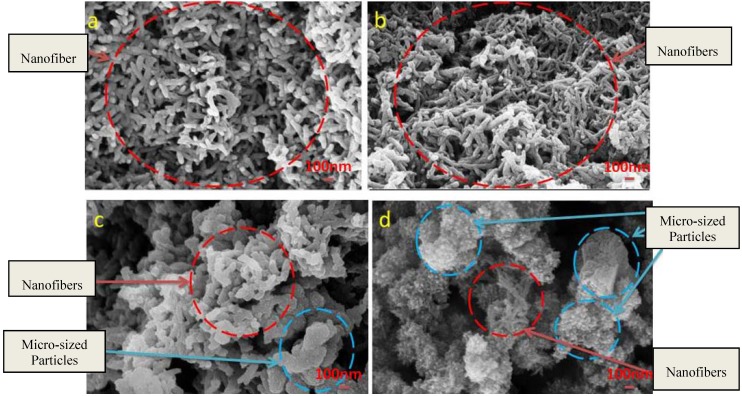
FESEM image of synthesized polyaniline by interfacial polymerization method with (**a**) 4.0; (**b**) 2.0; (**c**) 1.0; (**d**) 0.5 monomer/oxidant molar ratios.

Generally, three stages are proposed for polyaniline production: (1) nucleation; (2) initial growth; (3) secondary growth. In conventional methods, polyaniline nanofibers are formed initially due to the linear chain of polyaniline [22]. The formation of nanofibers takes place in nucleation and initial growth stages. Then the nanofibers act as nucleation centers for further polymerization of other aniline monomers (secondary growth stage). Actually, the secondary growth of polyaniline particles leads to formation of irregular shape of polyaniline in micro-size scale. In contrast in interfacial polymerization the final product appears in the form of nanofiber (Figure 1b). In interfacial polymerization, the initial formation of polyaniline nanofibers is not subjected to further polymerization. It is proposed that the secondary growth stage is suppressed, and only polyaniline nanofibers are generated. The rapid interaction of aniline monomer with oxidant and the fast movement of aniline monomer through water based solution leads to no aniline monomers supplied after the formation of PANI nanofibers, and only nanofibers are formed without further growth. Thus, the significant key to fabricate nanofibers in interfacial polymerization is to suppress the secondary growth stage [18]. The control in diameter and uniformity of polyaniline nanofibers is important to get high-quality product for different applications such as sensor and anticorrosive coating. It is found that the more uniform and thinner polyaniline nanofibers have better performance compared to non-uniform and thicker nanofibers [23,24]. Different parameters are responsible to control the morphology of polyaniline such as type of acidic dopant and oxidants. Another important factor which might influence on the morphology of the polyaniline nanofiber is the aniline/oxidant molar ratio. As shown in Figure 3 the synthesized polyaniline nanofibers with different aniline/oxidant molar ratios are produced via interfacial polymerization.

From the FESEM results in Figure 3, it is found that by decreasing the molar ratio from 4.0 to 0.5, the polyaniline nanofibers tend to agglomerate and form micro size particles. As seen in Figure 3a when the aniline/APS molar ratio is 4.0, the more uniform nanofibers with a mean diameter of 90nm have been produced. By decreasing the molar ratio to 2.0, also uniform polyaniline nanofibers are produced with an average diameter of 100nm. However, by further decreasing of the molar ratio to 1.0, the larger polyaniline nanofibers are produced with a mean diameter of 110 nm, and some micro size particles appeared. When the molar ratio reached to 0.5, the polyaniline with high level agglomeration are formed and low amount of nanofibers were produced. In other words, the high quality of polyaniline nanofibers with a thin diameter could be obtained by lowering the monomer/oxidant molar ratio. 

### 3.2. FTIR Spectroscopy

**Figure 4:**
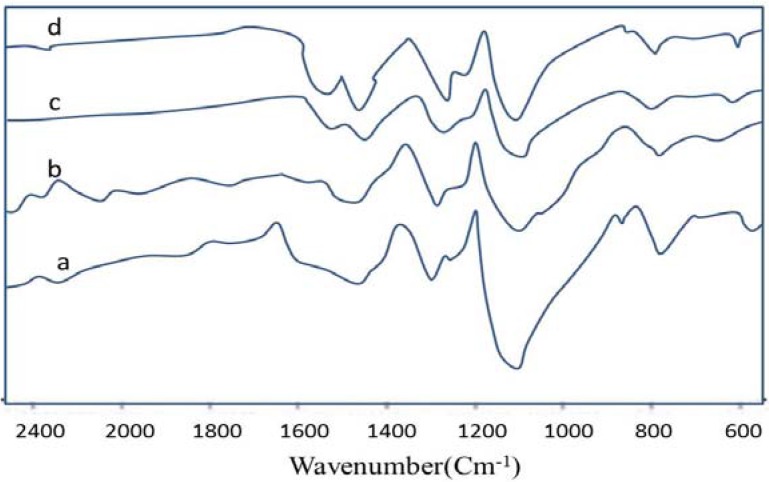
Fourier Transform Infrared Spectroscopy (FTIR) spectra of polyaniline nanofibers synthesized by interfacial polymerization at (**a**) 4.0; (**b**) 2.0; (**c**) 1.0; (**d**) 0.5 aniline/APS molar ratios.

Figure 4 shows the FTIR spectroscopy of the polyaniline synthesized by interfacial polymerization at different aniline/APS molar ratios.

As shown in the spectra of all samples the peaks are approximately presented at 802, 1240, 1300, 1490, 1570 cm^−1^. The main peaks at 1570 and 1490 cm^−1^ can be associated with C=N and C=C stretching vibrations of quinone and benzene rings, respectively. The peaks at 1300 and 1240 cm^−1^ are attributed to the C-N stretching mode. The peak at 1150 cm^−1^ is due to quinonoid unit of doped PANI. The peak at 802 cm^−1^ is attributed to the out-of-plane bending of C-H. These related peaks confirm the successfully formation of PANI at different aniline/oxidant molar ratios. 

### 3.3. XRD Analysis

**Figure 5:**
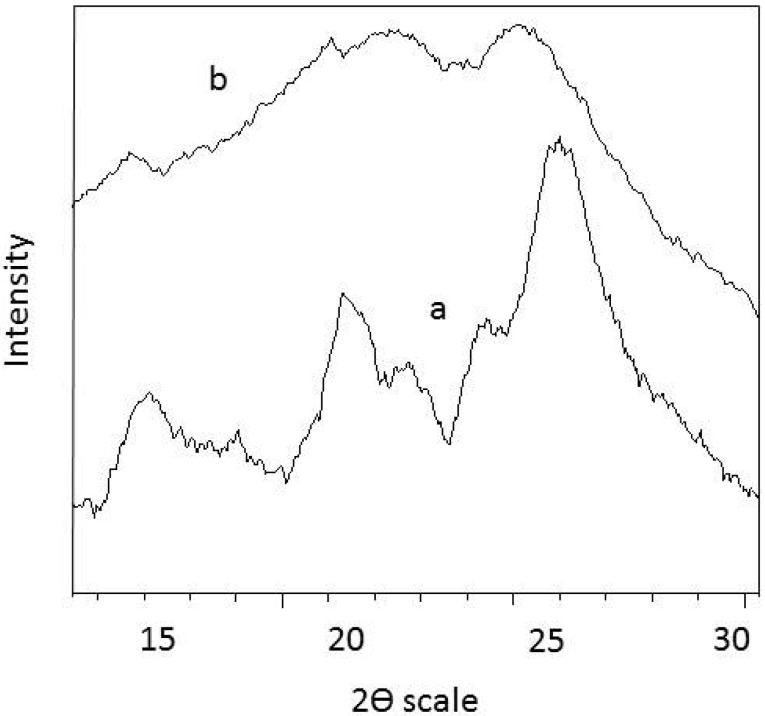
XRD analysis of (**a**) Polyaniline nanofibers obtained at aniline/oxidant = 4.0; and (**b**) irregular polyaniline micro-size particle obtained at aniline/oxidant = 0.5.

XRD analysis indicates the crystallinity of the product. High crystalline products could display metallic behavior and they are more useful than amorphous products. Generally, the ratio of half-width to height (HW/H) of an X-ray diffraction peak reflects the order of the polymer crystallinity. Rahy *et al.* [25] shows that the crystallined polyaniline has an X-ray diffraction pattern consisting of three peaks at 15°, 20° and 25°. As shown in Figure 5a three sharp peaks appeared at 2θ = 15°, 20.5°, 26°, reveal that polyaniline nanofiber with aniline/APS = 4 has crystalline properties.The characteristic peaks appeared at 15°, 20.5°and 26°, corresponding to (011), (020) and (200) crystal planes of PANI. As shown in Figure 5b, the broad peaks at 2θ = 20°, 25° reveal that polyaniline with aniline/APS = 0.5 is in the amorphous state.

## 4. Conclusions

PANI nanofibers were successfully prepared chemically by an interfacial polymerization. It has been found that different aniline/APS molar ratios affected the uniformity and morphology of polyaniline. The more uniform and thinner polyaniline nanofibers have been achieved at aniline/APS molar ratios = 4.0. XRD results show that polyaniline nanofibers with more uniformity at aniline/APS molar ratios = 4.0 has higher crystallinity rather than irregular shaped polyaniline. FTIR results also show that the polyaniline products have similar molecular structure in different aniline/APS molar ratios.
